# Diabetic Cardiovascular Autonomic Neuropathy, the Handgrip Test and Ambulatory Blood Pressure Monitoring Parameters: Are There Any Diagnostic Implications?

**DOI:** 10.3390/jcm9103322

**Published:** 2020-10-16

**Authors:** Miklós Kempler, Noémi Hajdú, Zsuzsanna Putz, Ildikó Istenes, Orsolya Vági, Magdolna Békeffy, Karolina Schnabel, Péter Kempler, Anna E. Körei

**Affiliations:** 1 Department of Internal Medicine and Haematology, Semmelweis University, Szentkirályi street 46, 1088 Budapest, Hungary; 2 Department of Internal Medicine and Oncology, Semmelweis University, Korányi Sándor street 2/a, 1083 Budapest, Hungary; hnola13@gmail.com (N.H.); zsuzsannaputz@yahoo.com (Z.P.); istenes.ildiko@med.semmelweis-univ.hu (I.I.); vagiorsi@gmail.com (O.V.); bekeffy.magdi95@gmail.com (M.B.); karolina.schnabel@gmail.com (K.S.); kempler.peter@med.semmelweis-univ.hu (P.K.); anna.korei@yahoo.com (A.E.K.)

**Keywords:** cardiovascular autonomic neuropathy, handgrip test, hypertension

## Abstract

Cardiovascular autonomic neuropathy (CAN) is a common complication of diabetes mellitus. Cardiovascular reflex tests (CARTs) are the gold standard in the diagnosis of CAN, but the handgrip test is no longer recommended to be performed. Previously, the inverse association between the presence of hypertension and handgrip test abnormality was demonstrated and hypertension as major cause for excessive diastolic blood pressure rise during handgrip testing in diabetic individuals proposed. The aim of the present study is to describe more precisely the association between handgrip test and hypertension by performing ambulatory blood pressure monitoring (ABPM) among diabetic patients. A more comprehensive evaluation of the relationship between cardiovascular autonomic function, hypertension and the handgrip test was targeted using heart rate variability (HRV) analysis. Our study involved 163 patients with diabetes. Cardiovascular autonomic neuropathy was assessed by the CARTs and sustained handgrip test was performed. All patients underwent ABPM and HRV analysis well. CAN was diagnosed in 69 patients. Significant associations were found between the diastolic blood pressure increase in response to handgrip exercise and the 24-h (rho = 0.245, *p* = 0.003), daytime (rho = 0.230, *p* = 0.005) and night-time (rho = 0.230, *p* = 0.006) mean systolic and 24-h diastolic (rho = 0.176, *p* = 0.034) blood pressure values, systolic blood pressure load (rho = 0.252, *p* = 0.003) and systolic (rho = 0.236, *p* = 0.005) and diastolic (rho = 0.165, *p* = 0.047) hyperbaric impacts. Higher values of ambulatory blood pressure monitoring parameters are associated with greater increases in diastolic blood pressure during isometric handgrip exercise. Diastolic blood pressure elevations during the handgrip test are also correlated, in order to diminished heart rate variability parameters attributable to parasympathetic dysfunction highlighting the pivotal role of sympathetic overactivity in evolving handgrip test results. Our study provides further evidence on the inverse association between handgrip test abnormality and hypertension in diabetic patients.

## 1. Introduction

Cardiovascular autonomic neuropathy (CAN) is a common complication of diabetes mellitus [1] and it has been reported to be associated with increased cardiovascular morbidity and mortality in diabetic patients [2,3].

Consequently, early and correct diagnosis of CAN cannot be overstated. Standard cardiovascular reflex rests (CARTs) are the most widely used in clinical practice. CARTs are safe, non-invasive, easy-to-perform tests and considered gold standard of CAN assessment [4]. Historically, a set of five standard CARTs including the handgrip test were used to assess CAN among diabetic patients. In recent guidelines, the measurement of diastolic blood pressure elevation in response to sustained handgrip exercise (handgrip test) has been no longer suggested to be performed [1,4]. However, there were no previous studies designed to judge the clinical relevance of the handgrip test and analysing its confounders in diabetic patients.

In our recent study involving over 350 patients with diabetes, we proved that sustained handgrip test has a poor sensitivity and specificity against the diagnosis of confirmed CAN and no associations with results of other CARTs could be demonstrated. Therefore, we provided evidence that the isometric handgrip test should no longer be applied for the evaluation of sympathetic dysfunction during cardiovascular autonomic neuropathy testing in diabetic patients. Variables confounding handgrip test results proved to be the presence of hypertension and the initial diastolic blood pressure values during handgrip exercise. More precisely, the presence of hypertension and handgrip test abnormality were inversely and independently associated [5] and this association was proposed as explanation for false negative handgrip test outcomes in diabetic patients with coexisting hypertension.

Recommendations agree that ambulatory blood pressure monitoring (ABPM) is essential for the diagnosis of white-coat-, nocturnal- and masked hypertension [6] and data from prospective studies with large number of patients indicate that day- and night-time blood pressure values are significant predictors of total and cardiovascular mortality, coronary artery disease and stroke. In addition, ABPM measures provide valuable data on the effectiveness of antihypertensive treatment [7]. Given the fact, that ABPM parameters not only allow precise characterization of hypertension but have powerful prognostic value for patients’ mortality as well as cardiovascular outcomes, they emerge as highly appropriate in more comprehensively assessing the relationship between hypertension defined by higher blood pressure values and sustained handgrip test. Moreover, a brief submaximal isometric handgrip test has recently been proposed as a tool to unmask masked-hypertension (diagnosed by ABPM) in a cohort of non-diabetic patients [8] indicating the potential diagnostic and prognostic role of the association.

Sustained isometric handgrip test was originally considered a test to detect diabetic cardiovascular sympathetic neuropathy. In contrast, the inverse association between the handgrip test abnormality and the presence of hypertension found lately [5] implies that the sympathetic overactivity accompanying hypertension might be equally or more important evolving handgrip test results. To clarify the relationship of the isometric handgrip test to autonomic parameters, heart rate variability (HRV) analysis was also performed. Heart rate variability analysis is considered one of the most sensitive approaches available for the evaluation of autonomic function [1]. In addition, HRV has been described as a predictor of future hypertension and cardiovascular disease as well [9,10].

Based on the above mentioned data, the present study aims to characterize more precisely the association between handgrip test and hypertension via performing ambulatory blood pressure monitoring (ABPM) among diabetic patients. Besides, we addressed the question of the relationship between cardiovascular autonomic function, hypertension and the handgrip test using heart rate variability (HRV) analysis.

## 2. Materials and Methods

The present cross-sectional study involved patients with type 1 and type 2 diabetes mellitus presenting at the Department of Internal Medicine and Oncology, Semmelweis University, Budapest, Hungary from March 2014 to 2017 December. Type 1 and type 2 Diabetes Mellitus (DM) were established according to the WHO (1999) criteria [11]. Patients hospitalized for acute intercurrent diseases (fever, infection, etc.) or for acute metabolic derangements were excluded from our study. Other exclusion criteria were diseases and conditions that may affect autonomic function, such as thyroid and liver diseases, chronic kidney disease, autoimmune or haematological disorders, neurodegenerative diseases, etc. Subjects with a history of arrhythmia, myocardial electrical conductance blocks, heart failure, ischaemic or valvular heart disease or pulmonary disorders (COPD) were also excluded. Further exclusion criteria included poor physical status of patients making them unable to exert sustained isometric muscular strain and the presence of proliferative retinopathy raising risk of intraocular haemorrhage during Valsalva and handgrip manoeuvre.

The study included 163 diabetic patients (69 male and 94 female) who were eligible to participate. Eligible subjects were asked to avoid strenuous physical exercise, caffeine beverages, tobacco products and alcohol in the 12 h prior to cardiovascular autonomic assessment. Patients on antihypertensive agents that might confound outcomes of CARTs based on heart rate changes were instructed to omit interfering medication, particularly beta-receptor blockers and non-dihydropyridine-type calcium channel blockers in the 24 h interval before cardiovascular autonomic testing. Patients were requested to follow their normal daily life avoiding any stressful condition and consume their normal usual diet in the days before, and during, testing.

All participants gave informed consent and the study protocol was approved by the local ethics committee. The datasets generated and analyzed during the current study are not publicly available due to its sensitive nature but are available from the corresponding author on reasonable request.

Data on age, glycaemic control (HbA1c), diabetes mellitus (DM) duration, antidiabetic and antihypertensive medication were acquired. Weight and height of eligible subjects were measured and body mass index (BMI) was calculated (expressed in kg/m^2^). Measurements of blood pressure (BP) values at rest and during the manoeuvres were performed using an OMRON M3 (Omron Corporation, Kyoto, Japan) automatic upper hand-cuff sphygmomanometer. Office BP values were obtained after a minimal period of 5 min resting state and the average of three seated BP measurements was calculated. Cardiovascular autonomic function was assessed by the 5 standard CARTs using Cardiosys 12.1 diagnostic station and Cardiosys-A01 software (MDE Heidelberg GMBH, Heidelberg, Germany). All tests were performed in a calm room according to standardised protocol [12]. Patients were all asked to consume only a light meal in the morning at least 2 h prior to testing and all patients underwent cardiovascular autonomic testing before noon. During real-time 12-lead monitoring recorded electrocardiography (ECG) signals were digitized at 2000 Hz sampling rate with a multichannel data acquisition system connected to a personal computer as described elsewhere [5].

Traditionally, CARTs based on heart rate variations (deep breathing test, Valsalva manoeuvre, lying-to-standing test) mainly reflect parasympathetic function, whereas those based on BP changes to manoeuvres (handgrip test and orthostatic hypotension test) characterize sympathetic function. The results of the deep-breathing test were expressed as the difference of the highest heart rate during inspiration and the lowest heart rate during expiration (beat-to-beat variation; beats/min). Valsalva ratio was assessed as the ratio of the longest RR interval after and the shortest RR interval during the manoeuvre. The results of the orthostatic hypotension test (OHT) were defined as the difference between the systolic BP obtained at rest in supine position and the lowest systolic BP after standing. 30:15 ratio during the lying-to-standing test was computed as the ratio of the RR intervals of the 30th and 15th (or nearby) sinus cycles following arising.

During the handgrip test, measurements were obtained with the dominant hand. Subjects were comfortably seated without any armrest with the shoulders being adducted and neutrally rotated and with the elbow at 90° flexion and the forearm and wrist in a neutral position in accordance with the recommendations of the American Society of Hand Therapists’ [13]. Firstly, maximal voluntary contraction was assessed. After that, subjects performed sustained handgrip exercise at 30% of maximal voluntary contraction up to 3 min. BP values were measured before the test (initial or baseline diastolic BP) and each minute in the 3-min period during sustained handgrip exercise. Diastolic blood pressure response/elevation (in mmHg) was calculated as the difference between the initial and the maximal diastolic blood pressure value measured during the test. To define normal versus abnormal handgrip test results (the latter also referred to as handgrip test abnormality), the traditional normative data of handgrip test defined by Ewing for diabetic patients were used [14] with the slight modification that all diastolic blood pressure elevations in responses to isometric handgrip exercise <16 mmHg were considered as abnormal. It means that diastolic blood pressure elevations of 11–15 mmHg previously considered as borderline were also included in the abnormal reference range in this study.

To evaluate parasympathetic neuropathy, age-related reference values for heart rate based tests were applied [12]. The confirmed diagnosis of CAN was defined as the presence of ≥2 abnormal test results excluding the handgrip test as recommended by recent guidelines [1].

All patients underwent 24-h ambulatory blood pressure monitoring (ABPM) and ECG-recording as well. ABPM and HRV parameters were obtained using Meditech Cardiotens 1.34 device [15]. Patients were asked to continue with their daily life during ABPM and they were instructed to avoid heavy physical exercise. The cuff was placed on the non-dominant arm. Blood pressure was recorded every 20 min during daytime (from 06.00 to 22.00) and every 30 min night-time (from 22.00 to 06.00). Short (from 20 s to 5 min-long) ECG-strips were recorded providing about 6 h of ECG data for HRV analysis. Only normal-to-normal beats were analysed while artefacts were filtered out. All recordings were visually reviewed as well. The definitions and normal values of the ABPM parameters applied are in accordance with the latest European Society of Cardiology and European Society of Hypertension (ESC-ESH) recommendations [16].

As for HRV, frequency domain parameters were calculated from spectral analysis of normal-to-normal intervals using fast Fourier transformation; the low (LF: 0.04–0.15 Hz), the high (HF: 0.15–0.4 Hz) frequency components, the LF/HF ratio and total power (TP: 0.0–0.4 Hz) were evaluated.

### Statistical Analysis

The Kolmogorov-Smirnov test for normality was accomplished on all variables. Normally distributed variables are expressed as mean ±SD while non-normally distributed data are described as median/geometric mean and interquartile range where appropriate. Categorical data are reported as n (%).

For comparison between groups with and without handgrip test abnormality, Mann-Whitney U-test or paired t-test for continuous variables and χ^2^ test for categorical data were carried out based on the variable’s normality of distribution.

Associations between the results of the handgrip test and ambulatory blood pressure monitoring (ABPM) parameters were analysed using Spearman’s rank correlation (rho) as neither diastolic blood pressure elevations during handgrip test nor ABPM parameters could be characterized by a stable normal distribution. Partial correlation was used for identifying significant correlations after adjustment for confounding variables.

All analyses were performed using SPSS 25 software (IBM, New York, NY, USA). Statistical significance was defined as *p* < 0.05.

## 3. Results

The present study involved a total of 163 patients: 20 (13.2%) with type 1 and 143 (87.7%) with type 2 diabetes; 148 (more than 90%) of our diabetic patients had coexistent hypertension as well. In 125 diabetic patients with hypertension, the diagnosis of hypertension was well known from past medical history and these patients were taking antihypertensive medication. We had 23 diabetic patients, in whom the diagnosis of hypertension was established upon ambulatory blood pressure monitoring. This way, 23 patients out of 38 (60.5%) diabetic patients with no previous hypertension in their medical history at baseline turned out to have hypertension upon ABPM studies. The main clinical characteristics of the study population are summarized in Table 1.

Diagnosis of confirmed CAN was proven in 42.3% of the patients. Abnormal results of the deep-breathing test, the Valsalva ratio, the 30/15 ratio, the handgrip and orthostatic hypotension test were present in 63.8%, 31.9%, 11%, 41.1% and 14.7%, respectively.

To describe groups with normal and abnormal handgrip test results, these two groups were compared in respect of demographic and clinical features as well as cardiovascular autonomic and ABPM parameters. Patients with *normal* handgrip test results had significantly higher 24-h mean systolic (134 vs. 128 mmHg, *p* = 0.004), higher daytime (130 vs. 137 mmHg, *p* = 0.004), night-time (120 vs. 129 mmHg, *p* = 0.010) mean systolic blood pressure values and 24-h mean diastolic (74 vs. 70 mmHg, *p* = 0.030) blood pressure values when compared to patients with abnormal handgrip test results. Similarly, patients with no handgrip test abnormality had higher systolic (46.2 vs. 32.4, *p* = 0.009) and diastolic (18.8 vs. 12.5, *p* = 0.039) blood pressure load and systolic (224.1 vs. 122.5, *p* = 0.004) and diastolic (47.2 vs. 27.8, *p* = 0.082) hyperbaric impacts than patients with abnormal handgrip test results. There was a trend towards diminished values of systolic and diastolic diurnal indices in the patient group with normal handgrip test result, but when compared to patients with abnormal handgrip test results, the difference did not reach statistical significance (6.1 vs. 6.4, *p* = 0.857 and 10.6 vs. 11.4, *p* = 0.661, respectively). In contrast, patient groups with normal and abnormal handgrip test result did not show any difference regarding age, BMI, diabetes duration, glycaemic control (HbA1c), presence of CAN and results of the other cardiovascular reflex tests. Among the HRV parameters, only total power (TP) (1717.2 vs. 1264.7 *p* = 0.047) was significantly reduced in patients with normal handgrip test result (i.e., patients with higher diastolic blood pressure response to handgrip test) compared to those with handgrip test abnormality (Table 2).

Correlations between ABPM and HRV measures and diastolic blood pressure elevations (in mmHg) during the handgrip test were also studied. Diastolic blood pressure response to handgrip test correlated significantly with 24-h mean systolic (rho = 0.245, *p* = 0.026), with daytime (rho = 0.230, *p* = 0.005) and night-time (rho = 0.230, *p* = 0.006) mean systolic blood pressure and diastolic (rho = 0.176, *p* = 0.034) blood pressure values, with 24-h systolic blood pressure load (rho = 0.252, *p* = 0.003) and with systolic (rho = 0.236, *p* = 0.005) and diastolic (rho = 0.165. *p* = 0.047) hyperbaric impacts. As for HRV measures, HF (r = −0.161, *p* = 0.047) and TP (r = −0.190, *p* = 0.020), markers of parasympathetic function, showed weak but significant negative correlations to diastolic blood pressure response during isometric handgrip test (Table 3).

As most ABPM and some of the HRV parameters had significant associations with age, BMI and initial diastolic blood pressure values measured at the beginning of isometric handgrip exercise, multiple correlation analysis was performed with adjustment for these confounding variables. Partial correlations of diastolic response to handgrip exercise with ABPM parameters, as well as correlations with HF and TP remained significant after control for age, BMI and initial diastolic blood pressure as presented in Table 4.

## 4. Discussion

Our present study aimed to further assess the association between handgrip test, cardiovascular autonomic function and hypertension via performing ambulatory blood pressure monitoring, and heart rate variability analysis in diabetic patients.

Our study confirms the inverse association between handgrip test abnormality and hypertension among patients with diabetes. Higher values of ambulatory blood pressure monitoring (ABPM) parameters are associated with greater increases in diastolic blood pressure during isometric handgrip exercise. Furthermore, associations of handgrip outcomes with higher parameters of ABPM and with diminished HRV measures of parasympathetic autonomic function imply that results of the handgrip test are rather marker of sympathetic overactivity accompanying hypertension.

CAN is an early and frequent complication of diabetes mellitus carrying a poor prognosis. Its prevalence reaches from 16.6% to as high as 65% in different diabetic patient populations depending on age, diabetes type and duration [1,12,17]. In the present study involving mostly elderly type 2 diabetic patients, prevalence of CAN based on the presence of at least 2 abnormal cardiovascular reflex tests was 42.3% and the most sensitive test was the deep-breathing test being abnormal in 63.8% of our patients, which is in agreement with previous findings [1,2,17].

We could not find any association between handgrip test abnormality and the presence of cardiovascular autonomic neuropathy. Handgrip test results did not show any relationships to the results of the deep-breathing test, the Valsalva ratio, the 30/15 ratio and the orthostatic hypotension test widely used for the assessment of sympathetic dysfunction, either. As a conclusion, the present study provides further evidence that diastolic blood pressure elevation in response to sustained handgrip exercise has no associations with other measures of CAN and should not be part of clinical CAN assessment. We could also reaffirm the inverse association between handgrip test abnormality and hypertension the latter previously proposed as the main confounder [5] regarding the handgrip test results. It means that patients with higher blood pressure values during ABPM, due to hypertension, are less likely to have abnormal handgrip test results, i.e., they have greater rise in diastolic blood pressure in response to isometric exercise. Conversely, patients with lower blood pressure values or being normotensive have abnormal handgrip test outcomes (less diastolic blood pressure elevation than 16 mmHg) more frequently.

ABPM is a safe, non-invasive method for the assessment of hypertension with many of its parameters being associated with cardiovascular morbidity and mortality [7]. Moreover, 24-h ABPM is considered to be the gold standard for diagnosing masked-hypertension [18]. The prevalence of masked-hypertension ranges up to 20% in population-based studies [19] and it is associated with target-organ damage and poor prognosis [20]. In the present study, more than 60% of our diabetic patients with no hypertension at baseline could be diagnosed with hypertension upon ABPM reflecting that masked-hypertension is a highly common disorder in diabetic patients. There is growing evidence that non-diabetic patients with masked hypertension exhibit a considerably greater rise in both systolic and diastolic blood pressure than normotensive control subjects during isometric exercise testing [8]. In fact, the exaggerated blood pressure responses in masked-hypertension were obvious from the first minute of submaximal isometric handgrip exercise and rise in total peripheral vascular resistance (TPR) was similarly exerted by ‘true hypertensive’ patients. In that study, blood pressure elevations and TPR during handgrip exercise showed correlations with 24-h blood pressure, with daytime and night-time mean blood pressure values, central blood pressure and aortic stiffness [8]. The excessive increase in total vascular resistance during exercise in patients with hypertension is of prognostic value regarding future cardiovascular events and total mortality [21].

In the present study, patients with normal handgrip test result having (supra-) physiological blood pressure increase during short-time isometric handgrip exercise had significantly higher 24-h mean systolic, and diastolic blood pressure, systolic and diastolic systolic and diastolic blood pressure load and hyperbaric impacts, compared to diabetic patients with abnormal handgrip test result. Similar differences could be proven between groups in respect of daytime and night-time mean blood pressure values. Association with night-time blood pressures is of special clinical importance as they were reported as stronger predictor of cardiovascular events and mortality than daytime blood pressure [22].

A correlation analysis was performed to get a more robust analysis of the association between diastolic blood pressure response to handgrip test and ABPM. Diastolic blood pressure elevations showed significant correlations with the 24-h, daytime and night-time mean systolic and 24-h diastolic blood pressures, with systolic blood pressure load and systolic and diastolic hyperbaric impacts. These correlations remained significant after adjustment for age, BMI and baseline diastolic blood pressure. Moreover, associations between diastolic blood pressure elevations and daytime and night-time diastolic blood pressure gained significance after adjustment. Therefore, our data provide evidence on independent, quantifiable correlations between the results of the isometric handgrip test and ABPM parameters in patients with diabetes mellitus. Interestingly, no significant correlations with diurnal indices could be proven though there was a trend toward diminished indices in the group with normal handgrip test result (>16 mmHg diastolic blood pressure response to handgrip exercise). On the one hand, this phenomenon could be a sample-size effect. On the other hand, diurnal indices might represent a different aspect of hypertension than mean blood pressure, blood pressure load or hyperbaric impacts given the fact that diurnal indices characterize the patients’ dipping status.

According to our data, diastolic blood pressure response to isometric handgrip test correlated significantly negatively with HF and TP components of HRV analysis. HF and fluctuations in the overall variability (TP) are attributable to parasympathetic nervous system activity. These findings are in accordance with the theory that autonomic imbalance as a consequence of parasympathetic neuropathy and sympathetic overactivity may play a crucial role in the pathogenesis of hypertension in diabetic patients [23]. In this context, diastolic hyper-responsiveness during isometric handgrip exercise may be considered as a marker of diminished parasympathetic function and sympathetic overactivity. The lack of associations between handgrip test result and LF and LF/HF in our study can be explained by the fact that we used a 24-h ECG-recording for HRV analysis. In fact, spectral analysis of HRV can only provide accurate characterization of sympathetic activity if the analysis was performed with control for respiration.

Findings in non-diabetic patients with hypertension imply that increased exercise vasoconstriction was a result of enhanced sympathetic activity and an impaired local vasodilatation during exercise in the contracting muscle [24]. Microvascular dysfunction and parasympathetic impairment were previously proposed the common pathways to the development of hypertension and diabetes mellitus as proposed by Frontoni et al. [25]. Large epidemiological studies also suggest that autonomic dysfunction may predict the development of future hypertension and type 2 diabetes [10,26]. In this context, it is a more provoking idea that diastolic hyper-responsiveness during handgrip exercise could be considered an early sign of this common parasympathetic dysfunction. Recent data on enhanced blood pressure response during the handgrip test in prehypertensive states [27] may also support the predictive role of isometric handgrip test—at least in hypertension. Koletsos et al. [8] have recently proposed isometric handgrip test a valuable tool for identifying patients with systolic and diastolic masked-hypertension and enabling early diagnosis and management of hypertension in non-diabetic individuals. Similarly, we have found associations between handgrip test and ABPM parameters of hypertension. By contrast, our patients were all diabetic and the majority of them were diagnosed with CAN potentially influencing outcomes.

Such an easy-to-perform, quick and cost-effective test such as isometric handgrip test would be promising for screening of patients for hypertension and cardiovascular risk stratification. In the recent study performed among non-diabetic individuals [8], the first minute blood pressure response to isometric handgrip exercise could be correlated with target organ damage characterized by parameters of cardiac hypertrophy. We have also proven associations of handgrip test results with some parameters of left ventricular hypertrophy [28]. These data imply that diastolic blood pressure response to isometric handgrip exercise may be used for quick screening instrument to reveal patients with masked-hypertension in both diabetic and non-diabetic individuals and identify patients at high risk for hypertension-induced target organ damage and cardiovascular events. However, appropriately powered, targeted follow-up studies with clinically relevant outcome measures are missing, and our study was not designed to investigate this issue. It could speculate that diastolic blood pressure elevations during the handgrip test could be a result of the counteraction between hypertension-related sympathetic overactivity (hypertension considered as a comorbidity of diabetes), as well as diabetes-induced parasympathetic neuropathy. However, given that associations with CAN parameters were scarce, but the handgrip test result showed significant associations with the presence of hypertension [5] with ABPM parameters in this study and with some measures of left ventricular hypertrophy [28], the impact of hypertension seems to be decisive on handgrip test outcomes.

Our study has strengths and limitations. The strengths of the present study are the relatively large number of patients studied by ABPM and the adjustment for potential confounders, such as age, BMI and initial blood pressure values.

One limitation could be the inclusion of both type 1 and 2 diabetic patients. However, neither literature data nor our previous studies revealed differences in features or pathogenesis of neuropathy between type 1 and type 2 diabetes. The mixed study population comprising both normo- and hypertensive individuals with diabetes could be considered as further limitation. Nevertheless, the fact that our study involved a broad spectrum of normotensive diabetic patients, patients with hypertension and patients in whom hypertension could only have been diagnosed via ABPM studies (i.e., diabetic patients with masked-hypertension) should rather underline the general relevance of the associations. If our analysis were performed only in diabetic patients with hypertension, the associations between handgrip test and ABPM parameters might have been more pronounced. The high proportion of diabetic patients with treated hypertension might be considered as a limitation of the study. Therefore, the effect of antihypertensive medication cannot be excluded. Due to the cross-sectional design of our study, no conclusion on causal relationship between handgrip test result and hypertension could have been established.

## 5. Conclusions

In summary, we could confirm the inverse association between handgrip test abnormality and ambulatory blood pressure parameters (ABPM) of hypertension. Higher values of ambulatory blood pressure monitoring (ABPM) parameters are associated with greater increases in diastolic blood pressure during isometric handgrip exercise and hence handgrip test abnormality will be very uncommon among these patients. Our results might involve the simple conclusion that the results of a diagnostic method based on measurement of blood pressure could not be independent from the presence of hypertension and as a consequence, handgrip test cannot be used to evaluate cardiovascular sympathetic neuropathy in diabetic patients. Moreover, associations of handgrip outcomes with higher parameters of ABPM and with reduced HRV measures of parasympathetic autonomic function suggest that diastolic blood pressure elevation during handgrip test should rather be considered a marker of sympathetic overactivity accompanying hypertension. The associations between diastolic blood pressure response to isometric handgrip test and measures of hypertension and target organ damage in this, and other studies, point to potential diagnostic and prognostic role of isometric handgrip test. Further prospective studies are warranted to clarify the potential role of diastolic hyper-responsiveness during the handgrip test as a screening tool for hypertension and cardiovascular risk prediction in diabetic and non-diabetic patients.

## Figures and Tables

**Table 1:** Main clinical characteristics of the study population.

Parameters (*n* = 163)	
Mean age (years)	60.8 ± 13 *
Gender (male/female)	69 (42.3%)/94 (57.7%)
HbA1c	7.5 ± 1.5% (58.5 ± 14.0 mmol/mol) *
Diabetes duration (years)	11.8 ± 10.3 *
Type 1/type 2 diabetes	20 (12.3%)/143 (87.7%)
BMI (kg/m^2^)	30 (27; 34) ^#^
Hypertension (yes/no)	148 (90.8%)/15 (9.2%)
Antidiabetic medication Met/SUR/DPP4i/insulin/acarbose	75(46%)/39(24%)/3(1.8%)/81(49.8%)/22(3.5%)
Antihypertensive medication (*n* = 125) ACEi or ARB/CCB/diuretics/AG/CA	106 (65%)/50 (30.7%)/42(25.7%)/19(1%)/38 (23%)
Statin use	59/163 (36.2%)
Aspirin use	58/163 (35.6%)

* mean ± SD; ^#^ geometric mean (interquartile range); Met: metformin; SUR: sulfonylureas; DPP4i: dipeptidyl-peptidase 4 inhibitors; ACEi: ACE inhibitors; ARB: angiotensin receptor blockers; CCB: calcium channel blocker. diuretics included hydrochlorothiazide and indapamide. AG: alpha1-receptor antagonists. CA: central-acting agents.

**Table 2:** Comparison of variables between diabetic subjects with and without handgrip test abnormality (univariate analysis).

Variables in Subjects	Abnormal Handgrip Test Result (*n* = 67)	Normal Handgrip Test Result (*n* = 96)	*p*
Age (years)	61.1 ± 11.9	60.4 ± 14.3	0.933 *
BMI (kg/m^2^)	30.8 (28; 35)	29.7 (26; 33)	0.213
Diabetes duration (years)	11.2 ±9.2	12.3 ± 11.1	0.755 *
HbA1c (%)	7.8 ±1.3	7.3 ± 1.4	0.159 *
HbA1c (mmol/mol)	62 ± 12	56 ± 13	0.161 *
Confirmed diagnosis of CAN	23 (34.3%)	44 (45.9%)	0.167 ^#^
Deep Breathing (1/min)	8.2 (5; 11)	8.1 (6; 12)	0.883
Valsalva ratio	1.26 (1.16; 1.29)	1.23 (1.14; 1.30)	0.689
30/15 ratio	1.12 (1.07; 1.18)	1.14 (1.07; 1.23)	0.508
Orthostatic hypotension (mmHg)	8 (0; 14)	9 (0; 14)	0.281
24-h mean systolic blood pressure (mmHg)	128 (120; 137)	134 (126; 143)	0.004
24-h mean diastolic blood pressure (mmHg)	70 (64; 78)	74 (66; 82)	0.030
Daytime mean systolic blood pressure	130 (124; 139)	137 (127; 147)	0.004
Daytime mean diastolic blood pressure	71 (66; 79)	76 (68; 86)	0.61
Night-time mean systolic blood pressure	120 (112; 130)	129 (117; 138)	0.010
Night-time mean diastolic blood pressure	64 (57; 70)	67 (60; 73)	0.125
Systolic blood pressure load (%)	32.4 (12.3; 49.9)	46.2 (15.4; 72.4)	0.009
Diastolic blood pressure load (%)	12.5 (0; 18.7)	18.8 (1.9; 31.1	0.039
Systolic hyperbaric impact (mmHgxh)	122.5 (23.4; 165)	224.1 (58.8; 346.3)	0.004
Diastolic hyperbaric impact (mmHgxh)	27.8 (0; 48)	47.2 (2.4; 77.4)	0.082
Systolic diurnal index (%)	6.4 (2; 12.4)	6.1 (0.1; 12)	0.857
Diastolic diurnal index (%)	11.4 (4.7; 17.4)	10.6 (3.5; 18)	0.661
High frequency component of HRV (HF)	202.5 (82;434)	136 (78; 240)	0.083
Low frequency component of HRV (LF)	361.3 (199; 646)	280 (147; 540)	0.279
LF/HF	1.717 (0.9; 2.7)	2.06 (1.3; 3.5)	0.095
Total power (TP)	1717.2 (1022; 2773)	1264.7 (724; 2445)	0.047

Data are reported as mean ±SD or geometric mean/median and interquartile range. Between-group comparisons were carried out by Mann-Whitney U-test or two-sample *t*-test (*) where appropriate. ^#^ χ^2^—for categorical variables were used as indicated. Categorical data are reported as *n* (%). Abbreviations: BMI: body mass index; HbA1c: haemoglobin A1c; CAN: cardiovascular autonomic neuropathy; HRV: heart rate variability.

**Table 3:** Correlations between 24-h ambulatory blood pressure (ABPM) and heart rate variability (HRV) parameters and diastolic blood pressure elevations in response to sustained handgrip test.

ABPM/HRV Parameter	rho	*p*
24-h mean systolic blood pressure	*r* = 0.245	0.003
24-h mean diastolic blood pressure	*r* = 0.176	0.035
Daytime mean systolic blood pressure	*r* = 0.230	0.005
Daytime mean diastolic blood pressure	*r* = 0.141	0.087
Night-time mean systolic blood pressure	*r* = 0.230	0.006
Night-time mean diastolic blood pressure	*r* = 0.145	0.064
Systolic blood pressure load	*r* = 0.252	0.003
Diastolic blood pressure load	*r* = 0.156	0.061
Systolic hyperbaric impact	*r* = 0.236	0.005
Diastolic hyperbaric impact	*r* = 0.165	0.046
Systolic diurnal index	*r* = 0.017	0.839
Diastolic diurnal index	*r* = 0.046	0.572
High frequency component of HRV (HF)	*r* = −0.161	0.047
Low frequency component of HRV (LF)	*r* = −0.121	0.139
LF/HF	*r* = 0.107	0.193
Total power (TP)	*r* = −0.190	0.020

**Table 4:** Partial correlations between 24-h ambulatory blood pressure (ABPM) and heart rate variability (HRV) parameters and diastolic blood pressure response during sustained handgrip after adjustment for age, BMI and initial diastolic blood pressure values as confounders (multiple correlation analysis performed on variables of Table 4 being significant at level *p* < 0.1).

ABPM/HRV Parameter	*r*	*p*
24-h mean systolic blood pressure	*r* = 0.334	<0.0001
24-h mean diastolic blood pressure	*r* = 0.280	0.002
Daytime mean systolic blood pressure	*r* = 0.295	<0.0001
Daytime mean diastolic blood pressure	*r* = 0.301	<0.0001
Night-time mean systolic blood pressure	*r* = 0.296	<0.0001
Night-time mean diastolic blood pressure	*r* = 0.270	0.001
Systolic blood pressure load	*r* = 0.267	0.003
Diastolic blood pressure load	*r* = 0.197	0.03
Systolic hyperbaric impact	*r* = 0.306	0.001
Diastolic hyperbaric impact	*r* = 0.210	0.021
High frequency component of HRV (HF)	*r* = −0.199	0.029
Total power (TP)	*r* = −0.210	0.021
